# Ionomic Responses of Local Plant Species to Natural Edaphic Mineral Variations

**DOI:** 10.3389/fpls.2021.614613

**Published:** 2021-03-29

**Authors:** Chengming Zhang, Syuntaro Hiradate, Yoshinobu Kusumoto, Sayaka Morita, Tomoyo F. Koyanagi, Qingnan Chu, Toshihiro Watanabe

**Affiliations:** ^1^Research Faculty of Agriculture, Hokkaido University, Sapporo, Japan; ^2^National Institute for Agro-Environmental Sciences (NIAES), Tsukuba, Japan; ^3^Graduate School of Agricultural and Life Sciences, The University of Tokyo, Tokyo, Japan

**Keywords:** ionomic variation, plant species, ecological speciation, environmental stress, adaptive evolutions

## Abstract

Leaf ionome indicates plant phylogenetic evolution and responses to environmental stress, which is a critical influential factor to the structure of species populations in local edaphic sites. However, little is known about leaf ionomic responses of local plant species to natural edaphic mineral variations. In the present study, all plant species and soil samples from a total of 80 soil sites in Shiozuka Highland were collected for multi-elemental analysis. Ioniomic data of species were used for statistical analysis, representing 24 species and 10 families. Specific preferences to ionomic accumulation in plants were obviously affected by the phylogeny, whereas edaphic impacts were also strong but limited within the phylogenetic preset. Correlations among elements resulted from not only elemental synergy and competition but also the adaptive evolution to withstand environmental stresses. Furthermore, ionomic differences of plant families were mainly derived from non-essential elements. The majority of variations in leaf ionome is undoubtedly regulated by evolutionary factors, but externalities, especially environmental stresses also have an important regulating function for landscape formation, determining that the contributions of each factor to ionomic variations of plant species for adaptation to environmental stress provides a new insight for further research on ionomic responses of ecological speciation to environmental perturbations and their corresponding adaptive evolutions.

## Introduction

Almost all terricolous plants depend on soil as their source of mineral nutrients and trace elements, including both essential and non-essential elements, to assemble an elemental composition in living organisms, which are defined as ionome (Salt et al., 2008). As a result of elemental accumulation, the ionome is regulated by both genetic and environmental factors (Neugebauer et al., 2020). With the development of high-throughput elemental analysis methods, such as inductively coupled plasma–mass spectrometry (ICP-MS) and ICP-atomic emission spectrometry (AES), ionomic research has been significantly accelerated (Huang and Salt, 2016).

To maintain ionomic homeostasis while adapting to different environments, the uptake, transport, and accumulation of mineral elements in different plant species are usually conducted with high specificity in terms of preference. Therefore, different plant species exhibit significant differences in ionome profile in the whole plant or in organs, tissues, or cells, although they live under identical soil conditions (Broadley and White, 2012; White et al., 2012; Watanabe et al., 2015). Fundamentally, natural selection causes plant species to choose different mutational and evolutionary strategies to adapt to environmental stresses, as reflected in the differences in their genomes and gene expression (Neugebauer et al., 2020). Because of limited resources in a small soil ecological environment, there may be overlapping niches among different plant species, leading to survival mode, with coexisting competition and cooperation (Treurnicht et al., 2019). Thus, the ionome of abundant plant species in a limited ecological system is largely influenced by the mineral nutritional status of soil and the considerable differences among plant species of contrasting nutrient acquisition strategies (Hayes et al., 2014). Generally, several plant species still have a high affinity to non-essential elements, although the quantity of essential elements in plants is much larger than that of non-essential ones (Watanabe et al., 2006). For example, *Melastoma malabathricum* was reported to hyperaccumulate aluminum (Al) combined with other nutrients in plant tissues to stimulate root activity, whereas the growth of barley was restricted with Al treatment (Watanabe et al., 2005). Furthermore, Praveen et al. (2019) planted wheat with several arsenic (As) accumulators (*Pteris vittata*, *Phragmites australis*, and *Vetiveria zizanioides*) in As-contaminated plots to reduce As accumulation in wheat. Consequently, the correlations of ionomic variations in plant species are important for ecological studies to understand how differences in soil mineral status affect elemental compositions in different plant species (Lai et al., 2018; Roeling et al., 2018; Pillon et al., 2019).

Plants living in different soil environments prefer to adjust their ionomic uptake and accumulation strategies to maximize their functional ionomic status. Thus, changes in soil conditions cause variations in plant ionome even for plants of the same species (Watanabe et al., 2015; Jiang et al., 2018). Genetic expression and regulation in plants are stimulated by environmental signals. Busoms et al. (2015) concluded that local adaptation occurred between coastal and inland populations of *Arabidopsis Thaliana* species living <30 km from each other and that adaptation was driven by different salinity levels. In Southwest Australia, members of the family Proteaceae efficiently evolved in terms of phosphorus (P) utilization mechanism to adapt to P-deficient soil (Lambers et al., 2015). Thus, the ionome of plants growing under different soil conditions is crucial for broadening current knowledge on ecologic adaptation.

Previous studies have mostly focused on the response of plants with respect to a single factor, such as plant species or soil condition, to environmental stress; however, very few studies have been conducted to elucidate the ionomic responses of different plant species of a large population to edaphic mineral variations. Foliar ionome depends largely, but not definitely (He et al., 2010; Geng et al., 2011), on soil mineral conditions and availability (Mueller et al., 2010). Furthermore, fallen senesced leaves can be degraded and returned to the roots, which may strongly affect productivity and diversity of microflora and ecosystems by changing soil humus quality and decomposition rates (Grime, 2001; Kitayama et al., 2004; Wardle et al., 2009). For these notions, we analyzed integrated leaf ionome across species and different soil conditions to reflect the nature and strength of mineral nutritional status in the ecosystem (Grime et al., 1997; Güsewell et al., 2005). We sampled and measured the concentration of 25 elements in leaves of different plant species *via* ICP-MS. We analyzed the obtained data through multivariate statistical analysis. The present study aims to examine the correlation among plant species and soil conditions on ionomic variations to reveal the responses of different plant species to environmental conditions.

## Materials and Methods

### Plant Materials and Soil Sampling in the Sites

**FIGURE 1:**
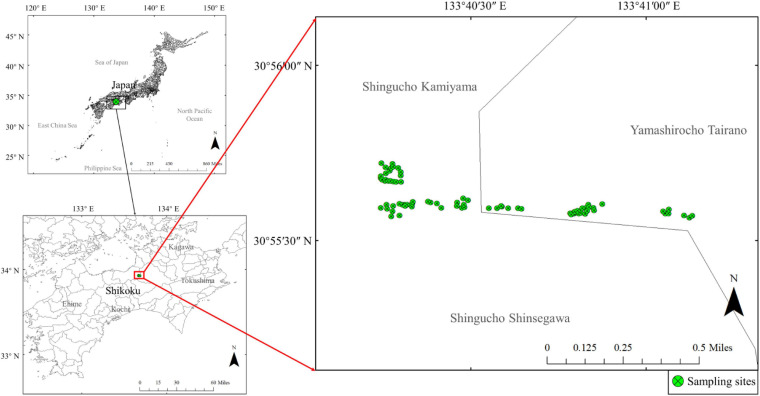
Study area and sampling site. The top-left map shows the location of Shikoku in Japan and the bottom-left indicates the location of Shiozuka highland. The main map on the right shows the distribution of sampling sites in the Shiozuka highland.

A field survey was conducted in Shiozuka Highland, Shikoku District, Japan (33°55′ N, 133°40′ E) (Figure 1). We examined a total of 80 sites, with a 1 m × 1 m quadrat in each site, within a 5-day period to avoid the potential effects of precipitation or temperature on ionomic variations. We sampled and recorded every plant species that were found in each site; however, only ionomic data of plant species found in more than 10 sites were subjected for subsequent analyses, which was represented by 24 plant species of 10 families (APG III, Smith et al., 2006; Supplementary Table 1). Plant samples were oven-dried at 70°C for 1 week and then ground into a fine powder using a vibrating cup mill made of zirconia (MC—4A, Ito Seisakusho, Tokyo, Japan). We collected soil samples from each site five times from a depth of 0 to 5 cm using a 100 mL cylindrical sampling core (5.0 cm high), and then, mixed them into one sample to represent the surface soil of each site, thereby eliminating edaphic imbalances.

### Ionomic Analysis of Samples

An approximately 200 mg aliquot of the powdered plant sample was added to 10 mL of approximately 60% HNO_3_ (specially prepared reagent; Nacalai-Tesque, Inc., Kyoto, Japan) in a glass digestion tube, stood overnight at room temperature, and then heated at 110°C with a DigiPREP MS apparatus (GL Sciences, Inc., Tokyo, Japan) until the leaf powder had almost disappeared. Then, we mixed the sample solution with 1 mL of H_2_O_2_ (semiconductor grade; Santoku Chemical, Tokyo, Japan) and heated at 110°C using the DigiPREP MS apparatus, which resulted in a clear solution. After cooling, the digested solution was filled to 50 mL using Milli-Q water, filtered using a filter paper (No. 5B, Toyo Roshi Kaisha, Ltd., Tokyo, Japan), and then subjected to ICP-MS analysis (Elan, DRC-e; PerkinElmer, Waltham, MA, United States) to determine the concentrations of 25 elements, namely, phosphorus (P), potassium (K), sulfur (S), calcium (Ca), magnesium (Mg), iron (Fe), manganese (Mn), zinc (Zn), copper (Cu), boron (B), molybdenum (Mo), nickel (Ni), Al, barium (Ba), sodium (Na), rubidium (Rb), strontium (Sr), As, cadmium (Cd), cobalt (Co), chromium (Cr), cesium (Cs), selenium (Se), lithium (Li), and vanadium (V). Air-dried soil samples were extracted using two extractants, namely, water (soil:Milli-Q water; 1:2.5, w/v) and 1 M ammonium acetate (soil:ammonium acetate; 1:5, w/v), for analysis. We directly measured the water extracts through ICP-MS, and ammonium acetate extracts were digested using the aforementioned procedure and then determined using ICP-MS. Available P was measured using the method previously described by Bray and Kurtz (1945). For the determination of soil pH, exchangeable acidity, and electrical conductivity, a multifunctional soil meter was conducted (Supplementary Table 2).

### Statistical Analysis

We conducted a linear mixed model using the residual maximum likelihood (REML) method to separate the estimated variance fractions of the total variance into plant species, soil sites, species–soil interaction, and residual components. The estimated means from the REML values were used as concentrations of individual elements to conduct a hierarchical cluster analysis (HCA). We performed a principal component analysis (PCA) using standardized (mean of zero and variance of one) leaf elemental concentrations in the plant families with the large ionomic differences in the results of HCA. Pearson’s correlation analysis was conducted at significance levels of *p* < 0.05 and *p* < 0.01. All statistical analyses were calculated using the Minitab 19 program (Minitab Inc., State College, PA, United States). We visualized data using multiple software, such as R (V.3.6.3) with “ggplot2” and “corrplot” packages, as well as TBtools (V.0.67) (Chen et al., 2020).

## Results

### Ionomic Profile of Different Plants Species Living in Different Soil Sites

**FIGURE 2:**
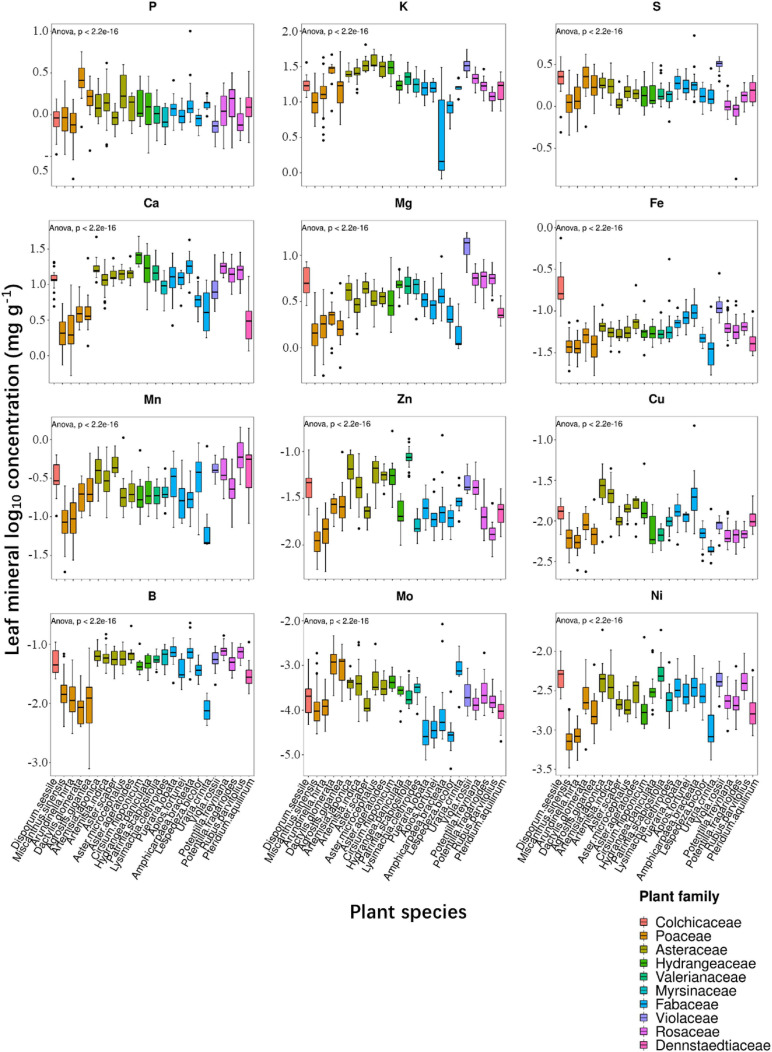
Boxplot showing leaf log_10_ concentrations of 12 essential elements (P, K, S, Ca, Mg, Fe, Mn, Zn, Cu, B, Mo, and Ni) of diverse plant species. Different box colors represent various plant families (as indicated below). Species from left to right: *Disporum sessile*, *Miscanthus sinensis*, *Arundinella hirta*, *Dactylis glomerata*, *Agrostis gigantea*, *Artemisia japonica*, *Artemisia indica*, *Aster scaber*, *Aster microcephalus*, *Aster ageratoides*, *Cirsium nipponicum*, *Hydrangea paniculata*, *Patrinia scabiosifolia*, *Lysimachia clethroides*, *Pueraria lobata*, *Apios fortunei*, *Amphicarpaea bracteata*, *Lespedeza bicolor*, *Uraria crinita*, *Viola rossii*, *Potentilla freyniana*, *Potentilla fragarioides*, *Rubus parvifolius*, and *Pteridium aquilinum*.

**FIGURE 3:**
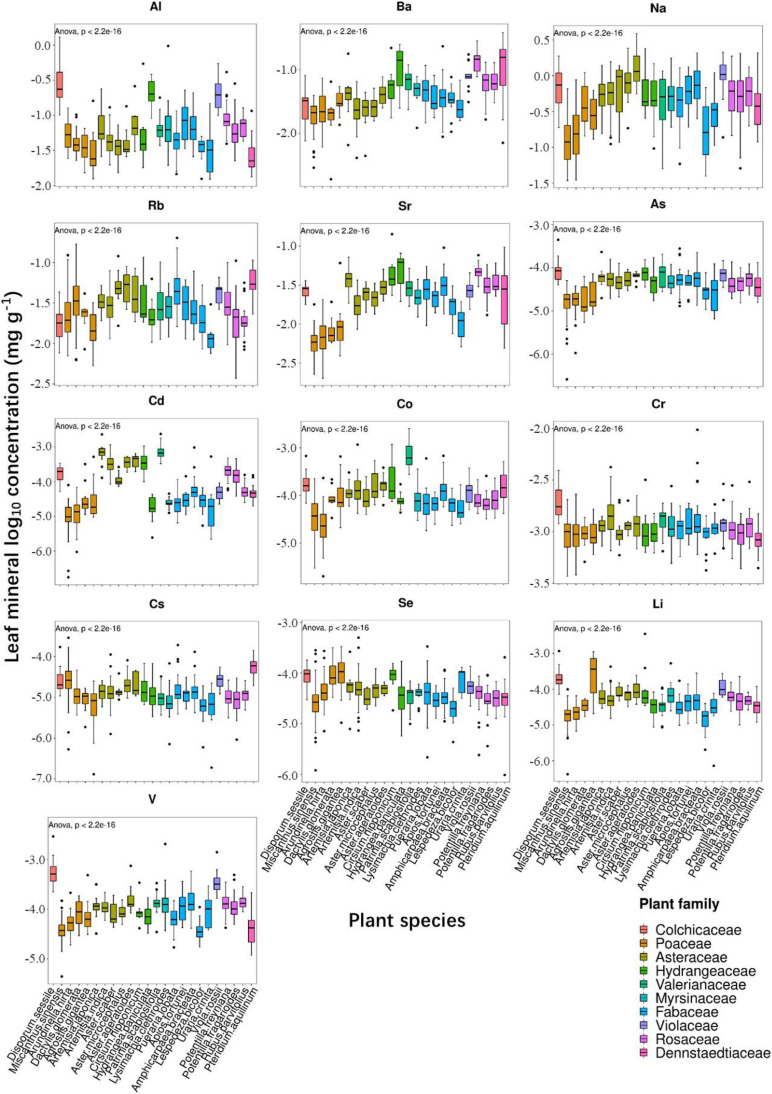
Boxplot showing leaf log_10_ concentrations of 13 non-essential elements (Al, Na, Ba, Rb, Sr, As, Cd, Co, Cr, Cs, Se, Li, and V) for 24 different plant species.

In the present study, we examined leaf ionomic profiles of plant species thriving in 80 different natural soil sites in Shiozuka Highland and finally selected 24 plant species belonging to 10 families that grow in more than 10 different sites for further analysis. The concentrations of nutrients and non-essential elements in plant leaves, respectively, as boxplots are shown in Figures 2, 3. For better visualization of the boxplots, we conducted a log_10_ conversion on raw ionomic data to avoid figure deformation caused by outliers. The concentration of elements in the leaves of different plant species followed an element specificity pattern of macroelements > microelements (Figures 2, 3). The concentration of each element in *Disporum sessile* was higher than that in *Miscanthus sinensis*, *Arundinella hirta*, *Dactylis glomerata*, and *Agrostis gigantea*, which belong to the family Poaceae, except for P, K, Mo, Ba, Rb, Cs, and Li. Furthermore, the leaf concentrations of most minerals in *D. glomerate* and *A. gigantea* were higher than those among other members of the family Poaceae. The concentration of most mineral elements was comparable among five plant species among the family Asteraceae, whereas the ionome of plant species among the family Fabaceae displayed a significant difference in several elements. For example, the concentrations of elements in the leaves of *Uraria crinita* were much lower than those in the leaves of other species among the family Fabaceae but contrastingly much higher in terms of Mo concentration. It demonstrated that there are different preferences for the accumulation of specific elements for different plant species, whereas species in the same family appear to have similar preferences for most elements.

### Ionomic Variations of Leaf Elements and Their Contributing Sources

The results of our descriptive statistical analysis that was conducted to quantify the sources of variation affecting each mineral element are presented in Table 1. The coefficient of variation (CV, the ratio of the standard deviation to mean) for leaf element concentrations listed was in a range from 0.44 to 2.40, increasing in the order S < K < Cr < P < B < Mg < Sr = Ca < Zn < Rb < Mn < Ni = Cu = Fe < As < Na < Se < Ba < Cs < Al < V < Cd < Co < Mo < Li. It indicated that non-essential elemental concentrations had larger variations than that of essential elements, excluding Cr (0.53), Sr (0.68), and Rb (0.74); and the CVs of macroelement concentration tended to be less than that of microelement concentration, except for Cr and Sr. The demand for essential elements, which is crucial to plant growth and development, turned out to be more stable than that of non-essential elements.

**TABLE 1:** Variations in leaf elemental concentrations among plant species and soil conditions (*n* = 549).

Leaf elements^*a*^	Mean	CV	REML (%)				*F* value^*b*^	
				
			Species	Soil	Interaction	Residual	*F*_*species*_	*F*_*soil*_
P	1.25	0.61	15.07	40.71	44.03	0.20	7.158**	5.356**
K	19.56	0.48	59.54	10.05	28.55	1.86	27.920**	1.370*
S	1.54	0.44	42.77	21.56	30.50	5.17	17.060**	2.748**
Ca	11.15	0.68	62.64	12.52	23.52	1.32	42.919**	1.721**
Mg	3.68	0.63	77.21	7.73	9.66	5.40	66.119**	1.314*
Fe	0.066	0.76	49.92	1.99	47.12	0.96	20.349**	0.891
Mn	0.28	0.75	38.38	18.87	0.05	42.70	19.521**	2.414**
Zn	0.032	0.72	66.79	8.32	0.11	24.78	42.523**	1.157
Cu	0.011	0.76	49.90	9.46	43.50	0.13	25.360**	1.197
B	0.050	0.62	50.12	18.56	31.12	0.10	30.296**	1.980**
Mo	0.32	1.84	12.19	52.64	34.39	0.78	6.600**	8.024**
Ni	0.0029	0.76	33.96	9.15	56.85	0.05	16.095**	1.402*
Al	0.079	1.27	45.02	4.06	49.74	1.18	15.267**	1.406*
Ba	0.054	0.96	52.57	6.96	40.45	0.03	29.329**	1.045
Na	0.56	0.82	34.40	19.34	46.25	0.00	11.517**	2.330**
Rb	0.034	0.74	18.03	39.37	29.97	12.63	5.882**	6.071**
Sr	0.025	0.68	56.05	9.60	31.45	2.91	33.437**	1.071
As	0.050	0.80	24.58	23.54	51.77	0.11	9.261**	2.792**
Cd	0.17	1.59	62.12	5.29	22.28	10.32	35.683**	1.392*
Co	0.14	1.64	50.64	9.75	37.35	2.26	23.686**	1.339*
Cr	1.19	0.53	14.50	35.15	37.98	12.36	4.686**	3.774**
Cs	0.020	1.25	18.77	18.60	58.98	3.65	7.238**	2.634**
Se	0.053	0.94	16.38	17.81	65.72	0.09	5.245**	2.939**
Li	0.075	2.40	27.97	0.61	71.41	0.01	6.953**	0.783
V	0.13	1.54	42.41	1.19	56.39	0.01	14.263**	0.947

^*a*^Data are expressed as μg g^–1^ for P, K, S, Ca, Mg, Fe, Mn, Zn, Cu, B, Ni, Al, Ba, Na, Rb, and Sr, whereas extremely trace elements such as Mo, As, Cd, Co, Cr, Cs, Se, Li, and V are expressed as mg g^–1^. ^*b*^ ANOVA with Tukey test are conducted for *F* values of leaf element concentrations attributable to plant species (*F*_*species*_) and soil sites (*F*_*soil*_). * and ** represent *p* < 0.05 and *p* < 0.01, respectively.

The linear mixed model was conducted using the REML method to segregate variance components of the total variance into plant species and soil sites. Species explained 12.19–77.21% of the total variance in leaf elemental concentration, whereas soil sites and interaction among different sites and plant species explained 0.61–52.64% and 0.05–71.41%, respectively (Table 1). Species exerted the strongest effect on almost half of the elemental concentrations among the four variance components, including all macroelements, except for P. The >50% variance in leaf K, Ca, Mg, Zn, B, Ba, Sr, Cd, and Co concentrations in all sites may be due to plant species. Inversely, the residual component explained < 10% of the total variance in all elemental concentrations, except for Mn, Zn, Rb, Cd, and Cr, and had a stronger effect than other components only in that of Mn (42.70%; Table 1). Soil condition played a bigger role than species only in P, Mo, Rb, Cr, and Se concentrations. The variations of most leaf mineral concentrations in our survey may be due to the interaction between species and soil, with values > 20%, even up to 71%, in that of Li. Furthermore, results showed that there was no interactive influence by soil condition and plant species on the variations of leaf Zn and Mn concentrations (Table 1). The analysis of variance (ANOVA) analysis results showed that interspecific ionomic variations were extremely significant (*p* < 0.01), irrespective of whether it is for macroelements or microelements. By contrast, diverse soil conditions caused a weaker effect on variations of elemental concentration (Table 1), since only half of the elements showed extremely significant differences (*p* < 0.01).

### Ionomic Network Among Different Plant Species and Soil Environment

**FIGURE 4:**
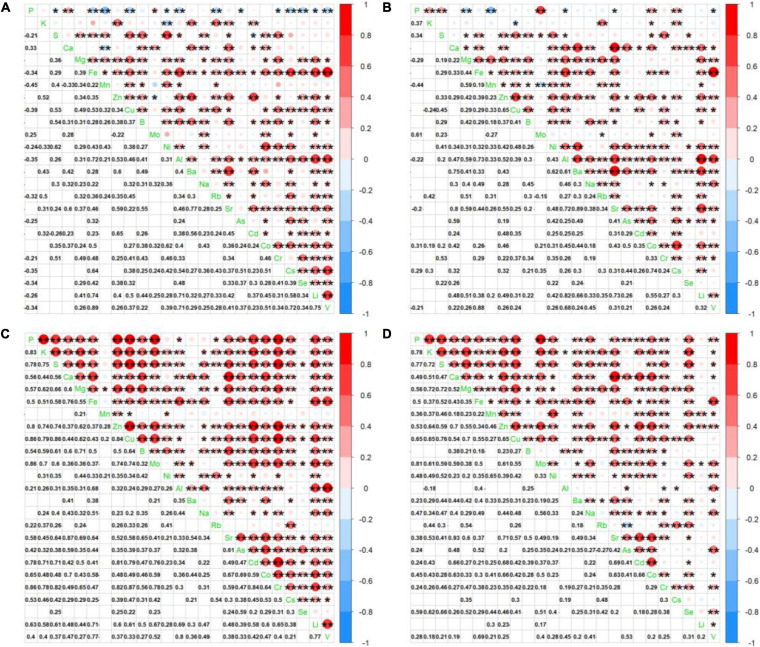
Heatmap showing correlation coefficients among leaf elements in the families **(A)** Rosaceae, **(B)** Asteraceae, **(C)** Fabaceae, and **(D)** Poaceae.

To verify whether significant interactions existed for leaf ionome among diverse plant species and soil conditions in the ecologic niche, we conducted correlation analysis. Due to the similarity of ionomic profiles in the same plant family (Figures 2, 3), we performed leaf elemental correlation analysis in different families. For a large sample size and higher statistical credibility, four families (Rosaceae, Asteraceae, Fabaceae, and Poaceae) with over three plant species in each family were representatively chosen to visualize in the correlation-heatmaps (Figures 4A–D). Generally, ionomic interactions in different plant families showed phylogeny-specific traits. Most of the minerals in the family Fabaceae displayed significantly positive correlations; however, that in other families expressed weaker correlations. Furthermore, ionomic correlations in the family Rosaceae showed several similarities to family Asteraceae, as well as those in the family Fabaceae to the family Poaceae. The macroelements in the families Rosaceae and Asteraceae have much weaker correlations with other elements than that in the families Fabaceae and Poaceae. However, correlations among the heavy metals in the family Asteraceae tended to be stronger than those in the family Poaceae, excluding Sr, As, and Cd. Interestingly, the interaction among several minerals in different families was variable, even completely opposite. For example, in the family Rosaceae, P was significantly (*p* < 0.05) negatively correlated with almost all other minerals, whereas it turned out to have a significantly positive correlation in the families Fabaceae and Poaceae. Therefore, different plant leaf ionomes are largely regulated by phylogenetic traits.

**FIGURE 5:**
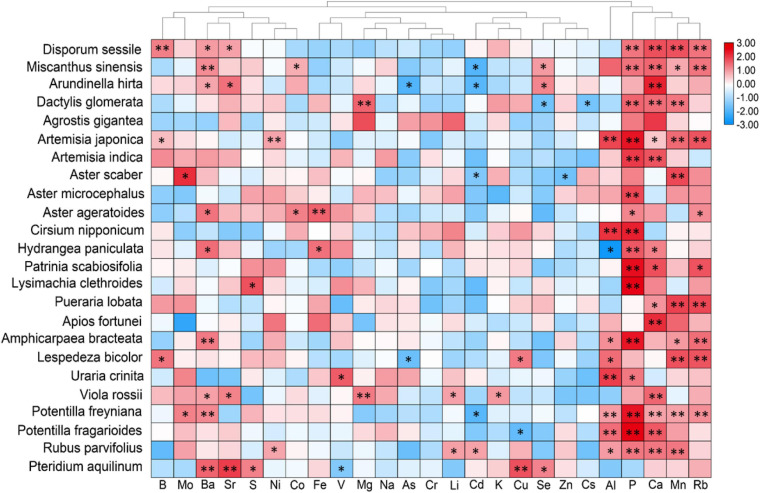
Heatmap showing correlation coefficients between leaves of different plant species and soil elemental concentrations. For soil elemental concentration data, P was shown as Bray II-P, and Soil B and Li were presented as a water-extractable form, respectively, whereas others were presented as a ammonium acetate-extractable form. Hierarchical cluster analysis was applied to minerals but not to plant species. Row data were normalized to fit the size relations of the correlation coefficients (*r*) for better visualization. The *r* was evaluated through Pearson’s correlation analysis. * and ** represent *p* < 0.05 and *p* < 0.01, respectively.

Furthermore, a correlation analysis between plant leaf and rooted soil minerals was conducted to better explain the response of leaf ionomic variations in plants to the effects of soil mineral status (Figure 5). Since the number of different plant species from different soils was not uniform, the correlation coefficient values of diverse species cannot be directly compared using the heatmap. We marked the significant correlation markers (^∗^ and ^∗∗^) in Figure 5 to aid our data interpretation. Hierarchical cluster analysis (HCA) results showed that the concentrations of Al, P, Ca, Mn, Ba, and Rb in soil showed significant correlations with those in most plant species. However, the significant correlations between most elements in leaf and soil were shown in only a few plant species, either for essential elements, such as K and Mg, or for non-essential minerals. For Na and Cr, no significant interaction was detected between leaf and soil in all species. Generally, the interactions between most minerals in leaf and soil were unremarkable. Interestingly, the leaf concentration of several heavy metals (Al, As, Cd, Cs, Se, V, Cu, and Zn) was notably negatively correlated with that in soil (Figure 5). It demonstrated that in the presently surveyed plant species, the phylogenetic traits can largely resist the variations of required soil minerals, but it may poorly adapt to fluctuations of soil P, Ca, and Mn concentrations.

### Ionomic Differences in Diverse Plant Species and Soil Sites

**FIGURE 6:**
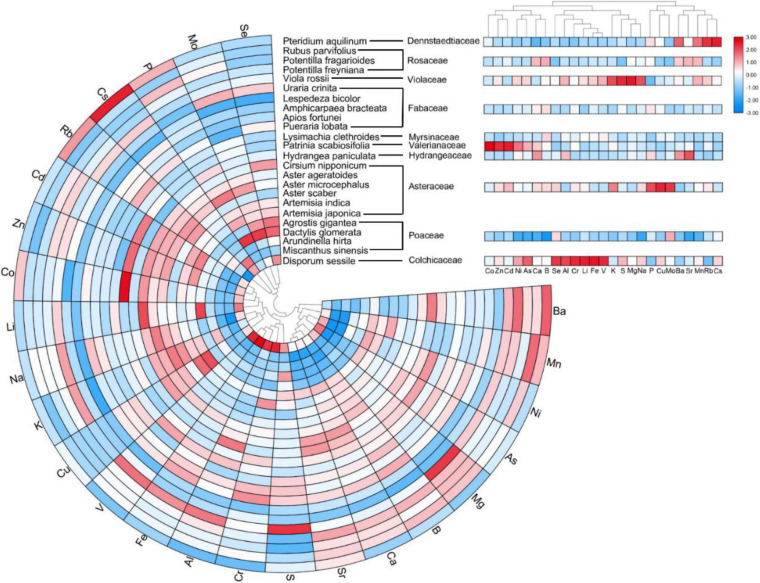
Composite loop heatmap showing ionomes of leaf elemental concentrations in diverse plant species with their respective families. Hierarchical cluster analysis was applied to minerals but not to plant species. For better visualization, a log_10_ transformation was conducted on raw data. Column data in plant species and families of different elements were normalized.

**FIGURE 7:**
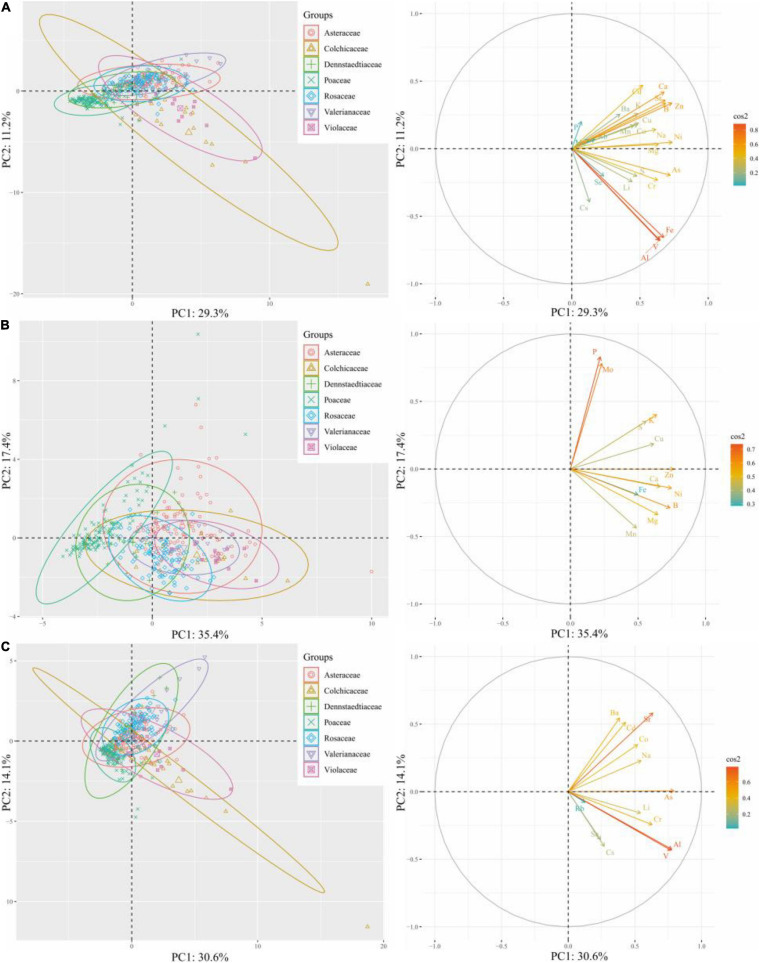
Principal component analysis (PCA) of elements in the leaves of plant species from different families. Scores on PC1 and PC2 with **(A)** all elements, **(B)** essential elements, and **(C)** non-essential elements, as well as the corresponding loading plots, respectively, were included.

To determine ionomic differences, we conducted hierarchical clustering of the leaf mineral concentrations of plant species and families using the estimated means from the REML analysis, and then, we visualized the data as a combined looping heatmap; however, HCA was only conducted to minerals, but not to species, because of the better observed classification of the families (Figure 6). The minerals in the plant species were classified into two principal clusters: Cluster 1 contained microelements Rb, Cs, Mo, Se, and macroelement P, whereas Cluster 2 included other elements. Although there was no dendrogram conducted among plant species, we observed that species in the same family showed higher similarities for element accumulation. The species that accumulated more elements in Cluster 1 were *Aster microcephalus*, *Aster ageratoides*, *Artemisia japonica*, *Artemisia indica* (belonging to Asteraceae), *Pteridium aquilinum*, and *Dactylis glomerata*. According to family clusters, Cluster 1 contained Rb, Cs, Mo, and P, plus Cu, Ba, and Sr, without Se. It clearly displayed that for P, Cu, and Mo, the plants in the family Asteraceae showed high accumulation; and for Ba, Mn, Rb, and Cs, *Pteridium aquilinum* (of the family Dennstaedtiaceae) showed the highest accumulation. However, there is no high accumulation of P in the family Poaceae, contrary to *D. glomerata*. It indicated the affinity to P in other members of the family Poaceae is low. Leaf elemental accumulation in Cluster 2 was also observed to be diverse in different species and families. Roughly, Cu, Na, Li, Co, Zn, Cd, and macronutrient K were mainly accumulated in *Disporum sessile*, *Viola rossii*, and members of the family Asteraceae. Furthermore, *Patrinia Scabiosifolia* showed the highest affinity to Co among all the species that were surveyed. For S, Cr, Al, Fe, and V accumulation, *D. sessile* and *V. rossii* showed the highest accumulation, and the species in the family Fabaceae (*Apios fortune* and *Amphicarpaea bracteata*) ranked second. However, the leaves of *Lespedeza bicolor*, *A. hirta*, and *M. sinensis* appeared insensitive to mineral accumulation, and all elements maintained a low level among species, showing that the latter two species were totally different from the rest of the species in the family Poaceae. Because of this, ionomic accumulation in the family Poaceae showed the lowest value among all families. For Cluster 2 in family classification, ionomic accumulation greatly varied, and different families held specific preferences or exclusions to several elements (Figure 6). Generally, essential elements were not separated from non-essentials, and macronutrients were not segregated from micronutrients. Principal component analysis (PCA) was only conducted to classify several families with ionomic variations in HCA, including all (Figure 7A), essential (Figure 7B), and non-essential elements (Figure 7C). The score plot and loading plot were separately displayed. The separation of the families in the three plots was weakly conducted. Only the family Poaceae was separated from the families Valerianaceae and Violaceae in all and essential elements because it was formerly located on the negative axis of PC1 in the three plots (Figures 7A,B). However, in the loading plots, there was no element negatively loading on PC1. It indicated that the family Poaceae had no affinity to all elements, which is consistent with our HCA results (Figure 6). Furthermore, the plots of all elements were similar to those of non-essential elements, indicating that non-essential elements, rather than essential elements, dominantly contributed to the observed ionomic differences.

## Discussion

Soil is known as a heterogeneous entity with possible elemental variations found within short distances (Baxter and Dilkes, 2012). Hence, we conducted a sampling design that corresponds to soil and plants and used multiple mixed means to reflect real soil conditions as close as possible. A widely edaphic mineral concentration range (CV from 0.36 K to 2.36 Mo) observed in this geographic scale suggests that the purpose of the present study, that is, to evaluate natural ionomic variations of plant species under diverse soil conditions, is achievable (Supplementary Table 2).

The interspecific and intraspecific variations of elemental concentration are shown in Figures 2, 3. Ionomic response to edaphic conditions among families tended to vary for most elements; however, preferences of different species in the same family to minerals were not always similar. As a result of the intervention of soil properties, several outliers existed in almost all the elements that are used by plant species under diverse soil conditions, resulting in higher ranges of the CV of leaf elements; however, the magnitude of leaf elemental concentration still followed the range of element specificity, that is, macroelements > microelements, which is similar to several previous studies (White et al., 2012; Watanabe et al., 2016; Stein et al., 2017; Neugebauer et al., 2020). The ANOVA and REML analysis results showed the species that dominantly contributed to variances in K, Ca, Mg, Fe, Cu, Zn, B, Ba, Sr, Cd, and Co concentrations, of which there is no significant difference among diverse soil sites in terms of Fe, Cu, Zn, Ba, and Sr concentrations (Table 1), which indicated more robust adaptation strategies to edaphic perturbations of these metallic elements than other elements, agreeing with the results of a study by White et al. (2012). Furthermore, leaf accumulation of heavy metals, such as Zn, Cd, and Pb, showed soil-independent population-related variations in *A. halleri* populations (Stein et al., 2017), which also supports the conclusion that the influence of soil to accumulation affinity to heavy metals is species-dependent (Xu et al., 2020). Contrastingly, soil factor contributed more than species to variations in P, Mo, Rb, Cr, and Se concentrations, especially more than 50% in Mo, and we coincidentally observed much higher variations in P and Mo concentrations in soil in the current survey (Supplementary Table 2). The same situation was observed in P, K, S, and Na in a previous study, with annual differential fertilizations (White et al., 2012). It demonstrated that high mineral concentration gradients of several elements in soil, to a certain extent, can perturb leaf ionome profiles. We also considered the species–soil interaction as a factor that is independent of residual components (Table 1). Most of the leaf mineral concentration variations in the present survey can be due to species–soil interaction, even up to 71% in that of Li. It indicated that ionomic variations represent the sum of ancient evolutionary occurrences in phylogeny and living environmental adaptations in ecosystems (van der Ent et al., 2018); hence, phylogenetic and environmental factors cannot be considered independent of each other in terms of affecting leaf ionome (Watanabe et al., 2006). Furthermore, because of the small geographic scale of our survey, the residual components that largely contributed to the findings in a previous study, such as latitude (Zhang et al., 2012), were small, except for Zn and Mn (Table 1). The real profiles of Zn and Mn in soil may be masked because of the strong heterogeneity of these elements, and results consequently underestimated the contributions of soil factors. In conclusion, for the main ionomic variation factor, there are considerable disagreements regarding this matter, as many researchers believed that species/families are the biggest driver for ionomic variations (Chu et al., 2015; Miatto and Batalha, 2016; Watanabe et al., 2016; Zhao et al., 2016), whereas (Zhang et al., 2012) and (Zhao et al., 2017) held the arguments that these variations were mainly driven by environmental conditions. Our results indicated that phylogenetic traits largely controlled the variations of leaf elemental concentration among plant species, whereas environmental impacts are limited within the preset range but still strongly affect variations of several elements.

Correlation analysis, as a common method for interaction (Feng et al., 2017; Du et al., 2020), was conducted to build the heatmap (Figures 4, 5). To stress the influences of phylogeny on ionomic interaction, correlations among elements were analyzed based on families. Several significant interactions were shown among elements in four plant families (Figure 4). For most elements, correlations showed similar positive/negative situations among families at different degrees. Elements with similar physicochemical properties are known to share or compete pathways or transporter systems to accumulate in leaves, as demonstrated in several studies on elements, such as Al and Fe (Watanabe et al., 2006), Cd and Zn (Baekgaard et al., 2010), As and S (Watanabe et al., 2014), As and Se (Zhou, 2017), and Cd and Se (Affholder et al., 2019). Interestingly, in the families Rosaceae and Asteraceae, the interaction between P and most of the other elements were significantly negative, whereas that in the families Fabaceae and Poaceae were positive. The *OsWRKY28* gene affects As (V) and P accumulation in rice by influencing phytohormone homeostasis (Wang et al., 2018). It is consistent with the relations between As and P in the families Fabaceae and Poaceae, whereas it was the opposite in the families Rosaceae and Asteraceae. In *A. thaliana*, roots and coumarin variation response to P deficiency were regulated by Fe homeostasis and vice versa (Chutia et al., 2019; Wang et al., 2019). However, in the family Rosaceae, the correlation between P and Fe was negative. It indicated that interactions between P and other elements were driven not only by mineral speciation but also by phylogenetic traits and environmental facilitation (Baxter, 2009); however, positive correlations consequently could not always be deduced for the same pathways (Baxter, 2009; Du et al., 2019). A low-P-tolerant species, *Hakea prostrata*, living in Southwest Australia was observed to have a similarly lower concentration of Zn and Cu under both hydroponic and soil conditions, whereas the concentrations of these metals in the hydroponics were much higher than in the soil conditions (Turner and Laliberté, 2015; Prodhan et al., 2017). *H. prostrata* was reported to take a strategy to reduce P use by controlling protein synthesis, leading to strict restrictions on Zn and Cu utilization (Prodhan et al., 2019). Therefore, we reasonably hypothesized that interactions among elements are regulated not only by pathway/transport systems but also by survival strategies to environmental stresses and genetic evolutions. The correlation analysis of elemental concentrations between leaf and soil provided a complementary explanation for ionome networks (Figure 5). Leaf P and Ca concentrations were significantly positively correlated to the corresponding minerals in the soil for most of the species; as well as leaf concentrations of Mn, Rb, Al, and Ba for more than five species (Figure 5). It is consistent with our previous analysis that soil contributed largely to P concentration variations in plant leaves (Table 1), supporting that leaf P concentration was strongly affected by soil conditions. However, as a robust mineral to soil variations, Ca also showed a strong positive correlation with soil Ca concentration without species disturbance. It is likely that the soil also has a strong effect on Ca concentration variations in leaves; however, species dominantly contributed to these variations. Notably, there were negative correlations observed in several species between leaf and soil for Cd, As, Cu, Se, Cs, and Al concentrations, respectively. A similar situation involving *Arabidopsis halleri* populations in heavy metal-contaminated sites exhibited lower Zn and Cd accumulation than *A. halleri* populations in non-metalliferous sites (Bert et al., 2002; Stein et al., 2017), which was interpreted as an outcome of natural selection by restricting plant growth and root activity, and this selection on heavy metals in leaf ionome seems more complex than other elements (Stein et al., 2017). It demonstrated that physiologic strategies of several species for hyper-tolerance were activated by heavy metal-polluted edaphic sites, implying adaptive evolution (Pauwels et al., 2012).

Hierarchical cluster analysis showed that the clustering of elements in species was similar to that in the families. Essential elements were not separated from non-essentials ones similarly that macronutrients were not departed from micronutrients at both species and family levels (Figure 6). According to HCA at the family level, minerals were clearly clustered and families were separated by ionomic profiles, which is consistent with many previous studies that distinguished plant families using their corresponding shoot or leaf ionomes (White et al., 2012; Watanabe et al., 2016; Neugebauer et al., 2020). However, PCA results did not perfectly support the conclusions, which may be due to the fact that only 50.5 and 44.7% of the total variance were explained by PC1 and PC2 in all essential and non-essential elements; hence, several separation information may be hidden by other dimensionalities. Most plant species strategically meet similar essential elements for growth and propagation, even those with extremely diverse preferences to non-essential elements and heavy metals (Marschner, 2012). Consistent with our PCA results (Figure 7), ionomic profile difference, to a certain extent, was driven by preference to non-essential elements.

As discussed, different plant species/families have different preferences and tolerances to soil properties. The competition of plants is imposed by natural selection to limit ecological niches, and only the fittest shall survive. Hence, we conducted the correlation analysis between vegetation cover ratio (VCR) and soil properties (Table 2). Results show that the VCR of *M. sinensis* showed significantly positive correlations for Al, Na, and other heavy metals but showed a negative correlation for P. Contrastingly, the VCR of *A. hirta* was negatively correlated with Al, and that of *A. indica*, with Na, Rb, and Se. It indicated that *M. sinensis* has stronger competitiveness to occupy ecological niches under acidic soil conditions than other competing species. Further research is warranted on the apoptosis/harvest of plants in the whole growth period under controlled regions to accurately study the ecologic niche competition driven by soil ionome.

**TABLE 2:** Correlation coefficients between vegetation cover ratios and soil extractable elemental concentrations^*a*^.

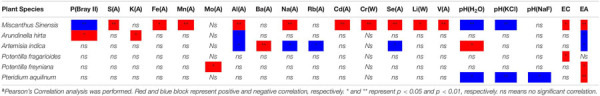

All in all, the present study complemented and provided a novel insight into the specific preferences that affect ionomic variations in plants, which were largely controlled by phylogenetic factors, whereas edaphic impacts were also strongly but limitedly (especially heavy metals) within the phylogenetic preset for species survival. Furthermore, these preferences and tolerances for minerals were ultimately translated into one of the determinants for plant survival under environmental stress conditions and in interspecific competition. Therefore, the present study presented new prospects and challenges for further research on ionomic responses of different plant species to environmental perturbations and adaptive evolutions.

## Data Availability Statement

The original contributions presented in the study are included in the article/Supplementary Material, further inquiries can be directed to the corresponding author/s.

## Author Contributions

TW, SH, and YK conceived of the presented idea. SH, YK, SM, and TK conducted the field work. CZ, TW, SH, YK, SM, and TK worked on the data collections and analysis. CZ, TW, YK, and QC wrote and revised this manuscript. All authors checked the results and contributed to the final manuscript.

## Conflict of Interest

The authors declare that the research was conducted in the absence of any commercial or financial relationships that could be construed as a potential conflict of interest.

## Supplementary Material

Supplementary Table 1 Plant species analyzed in this study.

Supplementary Table 2 Soil mineral element concentrations and chemical properties sampled from 80 different sites in Shiozuka Highland^*a*^.
