# Supplementary material for: Ionomic Responses of Local Plant Species to Natural Edaphic Mineral Variations
Source: Front Plant Sci. 2021 Mar 29;12:614613. doi: 10.3389/fpls.2021.614613 (PMC8039527; doi:10.3389/fpls.2021.614613)
Supplement: Supplementary Table 1 — Plant species analyzed in this study. [file Data_Sheet_1.pdf]

Table S1. Plant species analyzed in this study

Class	Order	Family	Species
Monocots	Liliales	Colchicaceae	*Disporum sessile* D. Don
	Poales	Poaceae	*Miscanthus sinensis* Andersson
			Arundinella hirta (Thunb.) Tanaka
			Dactylis glomerata L.
			Agrostis gigantea Roth
Asterids	Asterales	Asteraceae	*Artemisia indica* Willd.
			Artemisia japonica Thunb.
			Aster scaber Thunb.
			Aster microcephalus (Miq.) Franch. & Sav.
			Aster ageratoides Turcz.
			Cirsium nipponicum (Maxim.) Makino
	Cornales	Hydrangeaceae	*Hydrangea paniculata* Sieb.
	Dipsacales	Valerianaceae	*Patrinia scabiosifolia* Link
	Ericales	Myrsinaceae	*Lysimachia clethroides* Duby
Rosids	Fabales	Fabaceae	*Pueraria lobata* (Willd.) Ohwi
			Apios fortunei Maxim.
			Amphicarpaea bracteate (L.) Fernald
			Lespedeza bicolor Turcz.
			Uraria crinita (L.) DC.
	Malpighiales	Violaceae	*Viola rossii* Hemsl.
	Rosales	Rosaceae	*Potentilla freyniana* Bornm.
			Potentilla fragarioides L.
			Rubus parvifolius L.
Pteridopsida	Polypodiales	Dennstaedtiaceae	*Pteridium aquilinum* (L.) Kuhn

Table S2. Soil mineral element concentrations and chemical properties sampled from 80 different sites in Shiozuka Highland[a]

Soil property	Mean	Min	Max	SD	SE	CV
P (Bray II)	23.9	0.2	63.1	30.2	3.38	1.26
K (A)	287	109	585	103	11.53	0.36
S (A)	64	22	158	33	3.66	0.51
Ca (A)	1065	200	4930	787	88.02	0.74
Mg (A)	242	89	485	91	10.15	0.38
Fe (A)	4.93	0.89	14.9	2.80	0.31	0.57
Mn (A)	165	26	409	100	11.22	0.61
Zn (A)	1.03	0.18	3.34	0.58	0.064	0.56
Cu (A)	0.279	0.003	1.202	0.205	0.023	0.74
B (W)	0.040	0.003	0.161	0.280	0.031	0.70
Mo (A)	0.050	0.006	0.603	0.119	0.017	2.36
Ni (A)	0.13	0.02	0.31	0.06	0.0068	0.47
Al (A)	30.9	1.38	86.7	23.2	2.60	0.75
Ba (A)	20.8	6.70	45.2	9.00	1.01	0.43
Na (A)	24.8	4.89	65.1	13.0	1.45	0.52
Rb (A)	1.78	0.36	5.03	1.07	0.12	0.60
Sr (A)	2.83	0.73	7.38	1.47	0.16	0.52
As (A)	0.046	0.019	0.098	0.021	0.0023	0.45
Cd (A)	0.052	0.020	0.11	0.020	0.0022	0.38
Co (A)	0.134	0.090	0.264	0.056	0.0062	0.42
Cr (W)	0.033	0.011	0.062	0.011	0.0012	0.33
Cs (A)	0.058	0.008	0.155	0.034	0.0038	0.59
Se (A)	0.049	0.008	0.124	0.027	0.0030	0.55
Li (W)	0.0018	0.0001	0.0135	0.0019	0.0002	1.01
V (A)	0.038	0.009	0.085	0.016	0.0018	0.42
pH (H_2O)	5.11	4.54	6.26	0.42	0.047	0.08
pH (KCl)	4.10	3.61	5.41	0.41	0.046	0.10
pH (NaF)	8.16	7.21	9.61	0.47	0.052	0.06
EC (mS m^{-1})	23	11	148	15.4	1.72	0.68
EA ($mmol_c$ kg^{-1})	17.9	1.1	52.2	14.6	1.63	0.81

[a] Concentration values are placed as μg g^{-1} for all elements. A: ammonium-acetate-extractable form, W: water-extractable form, EC: electrical conductivity, EA: exchangeable acidity.
